# A Theranostic Small-Molecule Prodrug Conjugate for Neuroendocrine Prostate Cancer

**DOI:** 10.3390/pharmaceutics15020481

**Published:** 2023-02-01

**Authors:** Paulina Gonzalez, Sashi Debnath, Yu-An Chen, Elizabeth Hernandez, Preeti Jha, Marianna Dakanali, Jer-Tsong Hsieh, Xiankai Sun

**Affiliations:** 1 Department of Radiology, University of Texas Southwestern Medical Center, Dallas, TX 75390, USA; 2 Department of Urology, University of Texas Southwestern Medical Center, Dallas, TX 75390, USA; 3 Advanced Imaging Research Center, University of Texas Southwestern Medical Center, Dallas, TX 75390, USA

**Keywords:** prodrug conjugate, drug delivery, controlled drug release, theranostics, positron emission tomography (PET), targeted therapy, neuroendocrine prostate cancer, tumor innervation

## Abstract

After androgen deprivation therapy, a significant number of prostate cancer cases progress with a therapy-resistant neuroendocrine phenotype (NEPC). This represents a challenge for diagnosis and treatment. Based on our previously reported design of theranostic small-molecule prodrug conjugates (T-SMPDCs), herein we report a T-SMPDC tailored for targeted positron emission tomography (PET) imaging and chemotherapy of NEPC. The T-SMPDC is built upon a triazine core (TZ) to present three functionalities: (1) a chelating moiety (DOTA: 1,4,7,10-tetraazacyclododecane-1,4,7,10-tetraacetic acid) for PET imaging when labeled with ^68^Ga (t_1/2_ = 68 min) or other relevant radiometals; (2) an octreotide (Octr) that targets the somatostatin receptor 2 (SSTR2), which is overexpressed in the innervated tumor microenvironment (TME); and (3) fingolimod, FTY720—an antagonist of sphingosine kinase 1 that is an intracellular enzyme upregulated in NEPC. Polyethylene glycol (PEG) chains were incorporated via conventional conjugation methods or a click chemistry reaction forming a 1,4-disubstituted 1,2,3-triazole (Trz) linkage for the optimization of in vivo kinetics as necessary. The T-SMPDC, DOTA-PEG_3_-TZ(PEG_4_-Octr)-PEG_2_-Trz-PEG_3_-Val-Cit-pABOC-FTY720 (PEG_n_: PEG with n repeating ethyleneoxy units (n = 2, 3, or 4); Val: valine; Cit: citrulline; pABOC: p-amino-benzyloxycarbonyl), showed selective SSTR2 binding and mediated internalization of the molecule in SSTR2 ^high^ cells. Release of FTY720 was observed when the T-SMPDC was exposed to cathepsin B, and the released FTY720 exerted cytotoxicity in cells. In vivo PET imaging showed significantly higher accumulation (2.1 ± 0.3 %ID/g; *p* = 0.02) of [^68^Ga]Ga-DOTA-PEG_3_-TZ(PEG_4_-Octr)-PEG_2_-Trz-PEG_3_-Val-Cit-pABOC-FTY720 in SSTR2^high^ prostate cancer xenografts than in the SSTR2^low^ xenografts (1.5 ± 0.4 %ID/g) at 13 min post-injection (p.i.) with a rapid excretion through the kidneys. Taken together, these proof-of-concept results validate the design concept of the T-SMPDC, which may hold a great potential for targeted diagnosis and therapy of NEPC.

## 1. Introduction

A lethal disease with median survival of less than 1 year from the time of detection, neuroendocrine prostate cancer (NEPC) accounts for approximately 25% of all therapy- and castration-resistant prostate cancer (tCRPC) [1,2]. Since prostatic adenocarcinoma (ADPC) is a typical androgen-dependent (AD) cancer, androgen deprivation therapy is the standard of care for this disease. However, over the course of treatment, cancer cells can adapt and restore their growth signaling through mutated androgen receptors (ARs) or AR variants, necessitating the next therapeutic option—total androgen blockade. Nevertheless, the disease can further progress to become therapy- and castration-resistant, often exhibiting neuroendocrine features commonly known as NEPC—a subset of CRPC [2]. Without AR expression, the cells of tCRPC present low-to-zero prostate-specific membrane antigen (PSMA) [3,4,5]. Consequently, patients with NEPC cannot benefit from PSMA-targeted diagnosis and treatment options [6,7,8]. While the progression of NEPC is more aggressive, the lack of an understanding of its underlying biological mechanisms represents a challenge for the design of new therapies and diagnostic tools for the disease [9,10]. Currently an unmet clinical need, treatment options for patients with NEPC are limited [11,12].

Clinical diagnosis of NEPC relies on pathological examination of tissue biopsies, but the accuracy is subject to the heterogeneity of the collected tissue [13]. Given its noninvasive features and high sensitivity, positron emission tomography (PET) imaging with [^68^Ga]Ga-DOTATATE has been used in clinical practice for the diagnosis of patients with neuroendocrine tumors (NETs). In addition, a theranostic approach has been established to treat patients with [^177^Lu]Lu-DOTATATE [14]. Recently, radiolabeled antibody–drug conjugates (ADCs) have been reported for targeted therapy or imaging of NEPC [15,16,17]. However, ADCs suffer major drawbacks such as suboptimal in vivo distribution and kinetics, inefficient internalization into solid tumors (due to their large size), immunogenicity, irreproducible conjugation with drugs, and high manufacturing costs. In addition, the slow in vivo pharmacokinetics of monoclonal antibodies (mAbs) result in high radiation exposure and undesired side effects [18,19].

**Figure 1:**
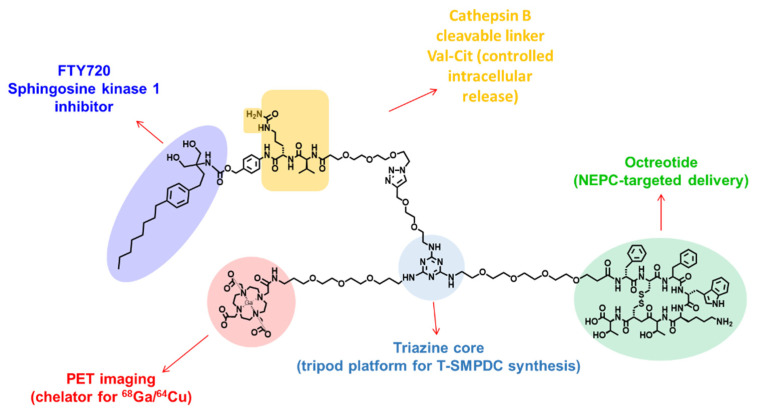
Design of a neuroendocrine prostate cancer (NEPC)-targeted chemotheranostic prodrug conjugate, DOTA-PEG_3_-TZ(PEG_4_-Octr)-PEG_2_-Trz-PEG_3_-Val-Cit-pABOC-FTY720. DOTA: 1,4,7,10-tetraazacyclododecane-1,4,7,10-tetraacetic acid; PEG_n_: polyethylene glycol chain with n repeating ethyleneoxy units (n = 2, 3, or 4); TZ: 1,3,5-triazine; Octr: octreotide; Trz: 1,4-disubstituted 1,2,3-triazole; Val: valine; Cit: citrulline; pABOC: p-amino-benzyloxycarbonyl; FTY720: fingolimod; T-SMPDC: theranostic small-molecule prodrug conjugate.

As such, we reason that small-molecule drug conjugates (SMDCs) can serve as an effective substitute for ADCs for the targeted delivery of chemodrugs due to their rapid distribution and clearance, good solid tumor penetration, non-immunogenic nature, definite chemical composition, and ease of synthesis. To date, we have reported two SMDC designs for the development of PSMA-targeted cancer theranostic agents [20,21]. The most recent one incorporated a prodrug linkage for controlled intracellular release of an immunomodulatory drug [20]. Herein, we further expand this theranostic small-molecule prodrug conjugate (T-SMPDC) design concept to develop a theranostic agent for NEPC-targeted delivery and controlled intracellular release of fingolimod (FTY720)—a sphingosine kinase 1 (SphK1) inhibitor—for precision therapy of prostate cancer (Figure 1). 

The T-SMPDC, DOTA-PEG_3_-TZ(PEG_4_-Octr)-PEG_2_-Trz-PEG_3_-Val-Cit-pABOC-FTY720, is built upon a 1,3,5-triazine core (TZ) to present three functionalities: (1) a chelating moiety (DOTA) for PET imaging when labeled with ^68^Ga (t_1/2_ = 68 min) or other relevant radiometals; (2) an octreotide (Octr) that targets somatostatin receptor 2 (SSTR2), which is overexpressed in the innervated tumor microenvironment (TME) [17,22,23,24]; and (3) FTY720 coupled with a cathepsin-B-cleavable linker comprising a valine–citrulline (Val-Cit) motif and a p-amino-benzyloxycarbonyl (pABOC) moiety. Polyethylene glycol (PEG) chains with a varying number of ethyleneoxy repeating units (n = 2, 3, or 4) were incorporated via conventional conjugation methods or a click chemistry reaction forming a 1,4-disubstituted 1,2,3-triazole (Trz) linkage for the optimization of in vivo kinetics as necessary.

It has been reported that chronic androgen deprivation therapy triggers the overexpression of Sphk1, which is implicated in the progression of NEPC [24,25]. A cytoplasmic kinase, SphK1 is a lipid mediator that is critical for tumor cell growth, survival, and therapeutic resistance. Therefore, SphK1 inhibitors have been used to suppress tumor growth [26,27]. The mechanism of action of FTY720—a SphK1 inhibitor—involves its phosphorylation in vivo to form fingolimod phosphate, with a similar structure to that of sphingosine 1-phosphate (S1P), which is the natural ligand of the sphingosine 1-phosphate receptors (S1PRs). However, while such inhibitory effects are desirable in cancer therapy, they might be detrimental to the fundamental biological processes regulated by S1PRs in normal cells. For instance, treatments of multiple sclerosis with FTY720 have been reported to show cardiovascular toxicity [28,29]. For this reason, targeted delivery and controlled release of FTY720 is of clinical significance to achieve an effective NEPC therapy while minimizing its toxicity to normal cells. Our design strategy is to introduce a cathepsin-B-cleavable linker between FTY720 and the conjugate platform.

To date, several lines of studies have reported upregulated levels of SSTR2 expression in NEPC [30]. For instance, the expression level of SSTR2 was found to be elevated following hormone depletion therapy [22], and high uptake of ^68^Ga-DOTATATE was observed in biochemically relapsed prostate cancer [1,31]. Most recently, targeting of SSTR2 was reported as a practical strategy to develop a polymeric nanoparticle system for NEPC therapy [32]. Therefore, we chose SSTR2 as a model NEPC target for the design of our T-SMPDC, DOTA-PEG_3_-TZ(PEG_4_-Octr)-PEG_2_-Trz-PEG_3_-Val-Cit-pABOC-FTY720. In this work, we present the T-SMPDC design along with its modular synthesis and the proof-of-concept biological evaluations of its potential application for NEPC-targeted diagnosis and chemotherapy.

## 2. Materials and Methods

### 2.1. General Materials and Procedures

All chemical reagents and solvents were purchased from commercial sources (Sigma-Aldrich, St. Louis, MO, USA; BroadPharm, San Diego, CA, USA; Fisher Scientific, Hampton, NH, USA). They were used as received unless stated otherwise. Milli-Q water was supplied by a Millipore Gradient Milli-Q water system (Burlington, MA, USA) and used for the preparation of all aqueous solutions. The characterization of the synthesized compounds, intermediates, and conjugates was performed on an Agilent 6540 Accurate-Mass Quadrupole Time-of-Flight Liquid Chromatography–Mass Spectrometry (LC–MS) system, which was coupled with a 1290 ultra-performance liquid chromatography (UPLC) system (Santa Clara, CA, USA). Unless otherwise noted, all LC–MS measurements were conducted in the positive ionization mode. Matrix-assisted laser desorption/ionization time-of-flight mass spectrometry (MALDI-TOF MS) was performed on a Voyager-DE PRO biospectrometry workstation (Applied Biosystems, Waltham, MA, USA) using 2,5-dihydroxybenzoic acid as the matrix. Nuclear magnetic resonance (NMR) spectra were recorded on a Varian 400 MHz spectrometer (Palo Alto, CA, USA). Purification of synthesized compounds was carried out using an Agilent 1260 Infinity preparative high-performance liquid chromatography (HPLC) system, which was equipped with a 1260 photodiode array detector (PDA) and an Agilent Prep-C18 column (150 × 21.2 mm, 5 µm) (Santa Clara, CA, USA). The radiolabeled compounds were characterized by an Agilent 1220 Infinity II analytical HPLC system equipped with a 1220 LC diode array detector (DAD) (76337 Waldbronn, Germany), an in-line Eckert & Ziegler radio detector (Eckert & Ziegler Radiopharma, Inc. Hopkinton, MA, USA), and a C18 Atlantis^®^ T3 column (4.6 × 250 mm, 5 μm) (Waters, Ireland).

### 2.2. Chemistry

For better clarity, the syntheses of compounds (**2**, **4**, **6**, and **8**) from their corresponding commercially available reagents (**1**, **3**, **5**, and **7**), along with their characterization data (Figures S1–S5), are presented in the Supplemental Materials.

#### 2.2.1. Synthesis of Val-Cit-pABOC-FTY720, Compound **10**

To the solution of azido-PEG3-Val-Cit-PAB-PNP (compound **9**, BroadPharm, San Diego, CA, USA) (9.2 mg, 0.01 mmol) in 3 mL of tetrahydrofuran (THF) and 1.5 mL of dimethylformamide (DMF) was added N,N-diisopropylethylamine (DIPEA) (38.7 mg, 0.3 mmol) at 0 °C. The reaction mixture was stirred for 15 min. Next, fingolimod, FTY720 (19.3 mg, 0.063 mmol) (Sigma Aldrich, Saint-Louis, MO, USA), which was previously dissolved in a mixed solution of 3 mL of THF and 1.5 mL of DMF, was added at 0 °C and stirred for 5 min under a nitrogen atmosphere. The reaction mixture was stirred at room temperature (r.t.) overnight. After solvent removal under reduced pressure, the crude mixture was purified by preparative HPLC (0 min: 10% CH_3_CN, 90% H_2_O; 20 min: 90% CH_3_CN; flow of 20 mL/min; all solvents contained 0.1% TFA). The pure fractions were evaporated and lyophilized to afford compound **10** (5 mg, 45%) as a tacky solid. MS (ESI) *m/z* calcd: 941.5586; found: 942.7114 ([M + H]^+^) (Figures S6 and S7). 

#### 2.2.2. Synthesis of TZ-PEG_2_-Trz-PEG_3_-Val-Cit-pABOC-FTY720, Compound **11**

To the solution of compound **10** (5 mg, 0.005 mmol) and compound **4** (0.3 mg, 0.001 mmol) in 1 mL of THF and 0.1 mL of DMF was added a solution of CuSO_4_ (0.35 mg, 0.002 mmol) in water and sodium L-ascorbate at r.t. under a nitrogen atmosphere. The reaction mixture was stirred at r.t. for 3 h. After solvent removal under reduced pressure, the crude mixture was purified by preparative HPLC (0 min: 10% CH_3_CN, 90% H_2_O; 20 min: 90% CH_3_CN; flow of 20 mL/min; all solvents contained 0.1% TFA). The pooled pure fractions were evaporated and lyophilized to afford compound **11** as a tacky solid (3.5 mg, 53.8% yield). MS (ESI) *m/z* calcd: 1231.5923; found: 1232.8495 ([M + H]^+^) (Figure S8).

#### 2.2.3. Synthesis of DOTA-PEG_3_-TZ-PEG_2_-Trz-PEG_3_-Val-Cit-pABOC-FTY720, Compound **12**

To the solution of compound **11** (5 mg, 0.004 mmol) in dichloromethane (DCM) and DIPEA at 0 °C was added compound **2** (3 mg, 0.003 mmol). The mixture was stirred for 7 h. After solvent removal under reduced pressure, the residue was purified by HPLC (0 min: 20% CH_3_CN, 20% H_2_O; 15 min: 50% CH_3_CN, 50% H_2_O; 20 min: 90% CH_3_CN; flow of 20 mL/min; all solvents contained 0.1% TFA) to yield compound **12** as a white powder (0.9 mg, 11% yield). MS (ESI) *m/z* calcd: 1970.1623; found: 1971.9273 ([M + H]^+^) (Figures S9 and S10).

#### 2.2.4. Synthesis of DOTA-PEG_3_-TZ(PEG_4_-Octr)-PEG_2_-Trz-PEG_3_-Val-Cit-pABOC-FTY720, Compound **13**

Compound **12** (2 mg, 0.001 mmol) was dissolved in a 1:1 solution of ethanol and 0.2 M borate buffer (pH = 8.5) to a final concentration of 2 mg/mL. Subsequently, octreotide-PEG_4_, compound **8** (6 mg, 0.004 mmol), was added. The resulting solution was stirred for 2 h. The crude mixture was purified by HPLC (0 min: 10% CH_3_CN, 90% H_2_O; 15 min: 55% CH_3_CN, 45% H_2_O; 20 min: 90% CH_3_CN; flow of 20 mL/min; all solvents contained 0.1% TFA) to obtain the desired protected product (1 mg, 33%). To the protected product, concentrated TFA was added, and the solution was stirred for 20 min. The reaction mixture was lyophilized to afford compound **13**. MS (MALDI-TOF) *m/z* calcd: 3031.5803; found: 1515.9345 ([M + 2H]^2+^) (Figures S11 and S12).

### 2.3. Radiochemistry

*Radiolabeling of DOTA-PEG_3_-TZ(PEG_4_-Octr)-PEG_2_-Trz-PEG_3_-Val-Cit-pABOC-FTY720 with ^68^Ga*: To an Eppendorf tube containing [^68^Ga]GaCl_3_ was added DOTA-PEG_3_-TZ(PEG_4_-Octr)-PEG_2_-Trz-PEG_3_-Val-Cit-pABOC-FTY720 (20 µg). The reaction mixture was incubated at 95 °C for 20 min at pH 3–3.5. After the incubation, the reaction was quenched with a solution of diethylenetriaminepentaacetic acid (DTPA) (10 µL, 10 mM), and the reaction mixture was diluted with DI water (15 mL) and loaded onto a preconditioned Waters Sep-Pak C18 short cartridge. The cartridge was washed with DI water three times (3 × 10 mL) to remove any unreacted [^68^Ga]. Air was used to remove any excess water from the cartridge, and the product, [^68^Ga]Ga-DOTA-PEG_3_-TZ(PEG_4_-Octr)-PEG_2_-Trz-PEG_3_-Val-Cit-pABOC-FTY720, was eluted from the cartridge using ethanol (0.5–1 mL).

*Radiolabeling of DOTA-PEG_3_-TZ(PEG_4_-Octr)-PEG_2_-Trz-PEG_3_-Val-Cit-pABOC-FTY720 with ^64^Cu*: To a solution containing 20 µg DOTA-PEG_3_-TZ(PEG_4_-Octr)-PEG_2_-Trz-PEG_3_-Val-Cit-pABOC-FTY720 in 100 µL of NH_4_OAc (0.4 M) buffer at pH 6 was added [^64^Cu]CuCl_2_ in 0.1 N HCl (approximately 0.296 GBq in 100 µL). The mixture was incubated at 70 °C for 40 min. The reaction was quenched with a solution of DTPA (10 µL, 10 mM), and the reaction mixture was diluted with DI water (15 mL) and loaded into a preconditioned Waters Sep-Pak C18 short cartridge, which was washed with DI water three times (3 × 10 mL), air-dried, and the product, [^64^Cu]Cu-DOTA-PEG_3_-TZ(PEG_4_-Octr)-PEG_2_-Trz-PEG_3_-Val-Cit-pABOC-FTY720, was eluted from the cartridge using ethanol (0.5–1 mL). 

### 2.4. Cell Culture and Animal Models 

All animal experiments presented in this work were approved by the Institutional Animal Care and Use Committee (IACUC) at the University of Texas Southwestern Medical Center (Dallas, TX, USA). Tumor-bearing animal models were established in the Hsieh Lab by following the animal protocol number (APN 2017-102131; three-year renewal approval date: 29 December 2022; expiration date: 29 December 2025). The mouse imaging procedures were performed according to APN 2020-102851 (approval date: 26 May 2020; expiration date: 26 May 2023) in the Sun Lab. The isogenic cell lines PC3_VC (SSTR2^high^) and PC3_sgPTP1B (SSTR2^low^) were generated in the Hsieh Lab. The parental PC3 cell line was purchased from ATCC (Manassas, VA, USA, CRL-1435); PC3_VC and PC3_sgPBP1B denote cells that were generated by CRISPR-vector and CRISPR-PTP1B gene knockout, respectively. Both cell lines were cultured in RPMI medium containing 10% fetal bovine serum, 1% penicillin/streptomycin, and 2 mM L-glutamine. The cells were harvested to establish the tumor model once reaching 75–80% confluency at 37 °C under 5% CO_2_. The dual-tumor murine model was developed by subcutaneously injecting 1.0 × 10^6^ cells in 100 µL of Hank’s Buffered Salt Solution containing 50% Matrigel into the shoulders of male severe combined immunodeficient (SCID) mice (NOD.CB17-Prkdcscid/NCrHsd, 6–8 weeks). The mice were allowed unrestricted access to autoclaved water and commercial diets and monitored closely for general observations and tumor burden assessment [20] throughout the entire period of the experiments.

For the cell-binding and internalization experiments, AR42J (SSTR2^high^) and PC3-Flu (SSTR2^low^) cells were used. AR42J cells were purchased from ATCC (Manassas, VA, USA, CRL-1492), while PC3-Flu cells were obtained from the laboratory of Prof. Martin G. Pomper at John Hopkins University (Baltimore, MD, USA). The subline PC3-Flu, from the androgen-independent PC3 human prostate cancer cell line derived from an advanced androgen-independent bone metastasis, was engineered to maintain no expression of PSMA according to reported procedures [33,34]. 

### 2.5. Cell Viability Assay

PC3 cells (3000–5000 cells/well) were seeded onto a 96-well plate. After one day, freshly prepared RPMI media containing different concentrations (0, 1, 5, 10 µM; n = 5) of FTY720 (Selleckchem, Houston, TX, USA) or compound **10** (0, 1, 3, 15, 30 µM; n = 5) were added to the cells, and the mixtures were incubated for 48 or 72 h. The relative number of viable cells was determined by an assay using 3-(4,5-dimethylthiazol-2-yl) -2,5-diphenyltetrazolium bromide (MTT) (Sigma-Aldrich, Saint-Louis, MO, USA), according to the manufacturer’s instructions, on a microplate reader at OD 570 nm. The experiment was repeated in triplicate, and data were represented as the mean ± standard deviation (s.d).

### 2.6. Cell Uptake Assay

The SSTR2-selective cell uptake assay was performed with [^64^Cu]Cu-DOTA-PEG_3_-TZ(PEG_4_-Octr)-PEG_2_-Trz-PEG_3_-Val-Cit-pABOC-FTY720 using SSTR2^high^AR42J and SSTR2^low^ PC3-Flu cells. Briefly, cells were seeded in a 24-well plate coated in polylysine (Nalgene, Rochester, New York, USA) (1.0 × 10^6^ cells per well, n = 3) and maintained for 24 h in a humidified incubator at 37 °C with 5% CO_2_. After then, the cells were incubated for 1 h at r.t. with [^64^Cu]Cu-DOTA-PEG_3_-TZ(PEG_4_-Octr)-PEG_2_-Trz-PEG_3_-Val-Cit-pABOC-FTY720 (~7.1 × 10^5^ CPM) in 500 µL of binding buffer (20 mM tris, 150 mM NaCl, pH 7.4). After removal of the solution, the cells were thoroughly rinsed three times with 500 µL of cold binding buffer and then solubilized with 500 µL of 1 M NaOH. The solutions were collected and counted using a PerkinElmer 2480 gamma counter (Richmond, CA, USA).

### 2.7. Internalization Assay

Approximately 3.0 × 10^5^ SSTR2^high^AR42J cells were seeded onto each well of a 6-well plate and maintained for 24 h in a humidified incubator at 37 °C with 5% CO_2_. After gently washing the cells with the binding buffer (20 mM tris, 150 mM NaCl, pH 7.4), a fixed amount (~5 × 10^5^ CPM) of [^64^Cu]Cu-DOTA-PEG_3_-TZ(PEG_4_-Octr)-PEG_2_-Trz-PEG_3_-Val-Cit-pABOC-FTY720 diluted with 400 µL of the binding buffer was added to each well. After incubation of the cells for 3, 10, 30, 60, 90, and 120 min (n = 3 each time point), the cells were thoroughly washed with ice-cold binding buffer for the removal of unbound [^64^Cu]Cu-DOTA-PEG_3_-TZ(PEG_4_-Octr)-PEG_2_-Trz-PEG_3_-Val-Cit-pABOC-FTY720. Then, the cells were incubated for 5 min with 0.5 mL of ice-cold stripping buffer (150 mM NaCl, 50 mM glycine, pH 3.0) to further remove surface-bound [^64^Cu]Cu-DOTA-PEG_3_-TZ(PEG_4_-Octr)-PEG_2_-Trz-PEG_3_-Val-Cit-pABOC-FTY720. After removal of the stripping buffer, the cells were lysed with 500 µL of 1 M NaOH (15 min at 37 °C). The solutions were collected for radioactivity counting on a PerkinElmer 2480 automatic gamma counter (Richmond, CA, USA) to quantify the internalized percentage (Internalized/Total Bound%) of [^64^Cu]Cu-DOTA-PEG_3_-TZ(PEG_4_-Octr)-PEG_2_-Trz-PEG_3_-Val-Cit-pABOC-FTY720. The experiment was repeated in triplicate, and data were represented as the mean ± standard deviation (s.d).

### 2.8. In Vitro Cathepsin-B-Mediated Release Assay 

A comparative cathepsin-B-mediated release assay was performed with compound **13** in the presence vs. absence of human cathepsin B (Sino Biological Inc., Chesterbrook, PA, USA). Briefly, compound **13** (150 µg) was dissolved in 60 µL of 100 mM citrate buffer (pH 5.5), followed by adding 300 µg of cysteine. Then, 20 µg of human cathepsin B in 40 µL of 100 mM citrate buffer was added to the mixture and incubated at 37 °C for 1 h. At the end of the incubation, the solution (20 µL) was sampled for HPLC and LC–MS analysis. Controls of this assay included compound **13** without being exposed to cathepsin B, as well as cathepsin B itself processed under the same conditions.

### 2.9. Small Animal PET/CT Imaging

The small animal imaging studies were performed when the size of the tumor xenografts reached the range 150–500 mm^3^ on a Mediso NanoScan PET/CT system (Mediso Medical Imaging Systems, Budapest, Hungary) equipped with a 4-mouse bed and a heating chamber to maintain the body temperature of the mice. The scans with [^68^Ga]Ga-DOTA-PEG_3_-TZ(PEG_4_-Octr)-PEG_2_-Trz-PEG_3_-Val-Cit-pABOC-FTY720 and [^68^Ga]Ga-PSMA-11 were arranged two days apart to ensure no signal interference between the two radiotracers. Before each scan, tumor-bearing mice were anesthetized and maintained under 1.5–2% isoflurane for the duration of the injection and scan. Immediately after intravenous injection of 3–4.4 MBq of each radiotracer, which was prepared in 100 µL of phosphate-buffered saline (PBS), a 60-min dynamic data acquisition was performed, followed by an anatomical reference CT scan. Using the Tera-Tomo 3D PET iterative algorithm with real-time Monte-Carlo-based physical modeling, we reconstructed the PET images into 10 frames of 1 min, 5 frames of 2 min, and 8 frames of 5 min. Quantitative imaging analysis was performed after the PET and CT images were co-registered to define regions of interest (ROIs) manually. The uptake of [^68^Ga]Ga-DOTA-PEG_3_-TZ(PEG_4_-Octr)-PEG_2_-Trz-PEG_3_-Val-Cit-pABOC-FTY720 or [^68^Ga]Ga-PSMA-11 in ROIs and tumor xenografts was calculated at each specific time of interest as the percentage of injected dose per gram of tissue (%ID/g).

### 2.10. Western Blot Analysis

Cell lysates of PC3_VC and PC3_sgPTP1B were prepared by lysing the cells on ice for 30 min using freshly prepared buffer, which contained 50 mM Tris-HCl (pH 7.5), 150 mM NaCl, 0.1% Triton X-100, 1 mM sodium orthovanadate, 1 mM sodium fluoride, 1 mM sodium pyrophosphate, 10 mg/mL aprotinin, 10 mg/mL leupeptin, 2 mM phenylmethylsulfonyl fluoride, and 1 mM EDTA. After centrifugation of the cell lysates at 14,000 rpm for 30 min at 4 °C, the protein extracts were loaded for sodium dodecyl sulfate–polyacrylamide gel electrophoresis (SDS–PAGE) using Bolt 4–12% NuPAGE gels (Life Technologies, Carlsbad, CA, USA) and then blotted onto a nitrocellulose membrane using the Trans-Blot Turbo Transfer system (BIORAD, Hercules, CA, USA). After the membrane was incubated in the solution of primary antibodies against PTP1B (ProteinTech, Rosemont, IL, USA) and SSTR2 (Abcam, Cambridge, UK) at 4 °C for 16–18 h, it was washed and then incubated with horseradish-peroxidase-conjugated secondary antibodies at r.t. for 2 h. The blotting results were read using an Advansta ECL chemiluminescent detection system (San Jose, CA, USA). The loading control was β-actin (Santa Cruz Biotechnology, Dallas, TX, USA).

### 2.11. Immunohistochemistry (IHC) 

Tumors were excised after the experiments and immediately fixed in 10% neutral-buffered formalin for 48 h. The tissue samples were processed and embedded in paraffin blocks. For IHC, the formalin-fixed, paraffin-embedded sections of the tumors were deparaffinized, rehydrated, and subjected to heat-induced antigen retrieval (citrate buffer, pH 6.0). After incubation with primary antibodies against SSTR2 (Abcam, Cambridge, UK) or PSMA (Abcam, Cambridge, UK), the sections were developed with 3,3′-diaminobenzidine chromogen, followed by counterstaining with hematoxylin and eosin. The representative photograph was taken with a Nikon microscope.

## 3. Results

### 3.1. Modular Synthesis of DOTA-PEG_3_-TZ(PEG_4_-Octr)-PEG_2_-Trz-PEG_3_-Val-Cit-pABOC-FTY720

**Scheme 1:**
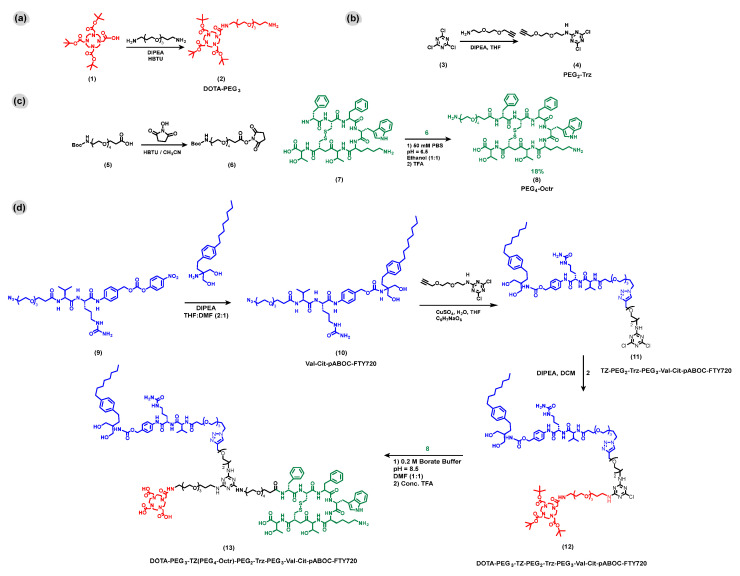
Synthetic route to DOTA-PEG_3_-TZ(PEG_4_-Octr)-PEG_2_-Trz-PEG_3_-Val-Cit-pABOC-FTY720. (**a**) DOTA chelator and PEG_3_ diamine linker condensation reaction. (**b**) Triazine core SN2 reaction with a propargyl-PEG_2_-amine group. (**c**) Activation of the succinimidyl-ester- PEG_4_ linker. (**d**) The FTY720 SN2 reaction with the cathepsin-B-cleavable linker followed by its addition to the triazine core through the propargyl-PEG_2_ linker using click chemistry, the DOTA-PEG_3_ (compound **2**) SN2 reaction with compound **11**, the octreotide-PEG_4_ molecule substitution reaction with the triazine core, and hydrolysis of the tertbutyl groups from the DOTA chelator to yield the designed T-SMPDC, DOTA-PEG_3_-TZ(PEG_4_-Octr)-PEG_2_-Trz-PEG_3_-Val-Cit-pABOC-FTY720, compound **13**. DIPEA: N,N-diisopropylethylamine; DMF: N,N-dimethylformamide; HBTU: (2-(1H-benzotriazol-1-yl)-1,1,3,3-tetramethyluronium hexafluorophosphate; TFA: trifluoroacetic acid, THF: tetrahydrofuran.

The modular synthesis of DOTA-PEG_3_-TZ(PEG_4_-Octr)-PEG_2_-Trz-PEG_3_-Val-Cit-pABOC-FTY720 was enabled by presenting three different functionalities on the backbone of a 1,3,5-triazine core, TZ (Scheme 1) [20,35,36,37]. The different functionalities were added to TZ through two different linkers. For the conjugation of the DOTA chelator and the octreotide to TZ, PEG chains were used because of their well-established roles in drug delivery [38,39]. For the conjugation of FTY720 to the molecular structure, a cathepsin-B-cleavable linker was used to enable the desired controlled prodrug release (Scheme 1a–d).

The multistep synthesis started with the preparation of each individual component, to enable their incorporation to TZ at later steps. A DOTA chelator (Scheme 1a) was added to a PEG_3_ diamine linker through a condensation reaction. For the scope of this work, the chelator was incorporated for radiolabeling the T-SMPDC with ^68^Ga or ^64^Cu for PET imaging or in vitro assays. The triazine core was functionalized with a propargyl-PEG_2_-amine group (Scheme 1b) through an SN2 reaction that functioned as the connecting site for the FTY720–cathepsin B conjugate. A succinimidyl-ester-activated PEG_4_ linker was synthesized to serve as a linker between the octreotide molecule and the triazine core (Scheme 1c). For the chemotherapeutic functionality, the FTY720 molecule was attached to the cathepsin-B-cleavable linker through an SN2 reaction, followed by its addition to the triazine core through the propargyl-PEG_2_ linker using click chemistry (Scheme 1d). For the imaging functionality, the DOTA-PEG_3_ (compound **2**) was then attached to the triazine core using an SN2 reaction between the chlorine of the triazine and the amine on the N-terminal of compound **2**. Lastly, to add the SSTR2-targeting functionality, the octreotide-PEG_4_ molecule was added to the triazine core through a substitution reaction. In the last step, the tertbutyl groups from the DOTA chelator were hydrolyzed to yield the designed T-SMPDC, DOTA-PEG_3_-TZ(PEG_4_-Octr)-PEG_2_-Trz-PEG_3_-Val-Cit-pABOC-FTY720 (Scheme 1d). Detailed reaction conditions and compound characterization for each step are provided in the Supplemental Materials (Figures S1–S12).

### 3.2. Radiochemistry 

DOTA-PEG_3_-TZ(PEG_4_-Octr)-PEG_2_-Trz-PEG_3_-Val-Cit-pABOC-FTY720 was radiolabeled with ^68^GaCl_3_ in a decay-corrected radiochemical yield of ~15%. The average molar activity was 38 GBq/µmol (n = 3). The ^64^Cu version of the conjugate was prepared for in vitro binding and internalization assays, which require a longer-lived radionuclide than ^68^Ga. The radiolabeling reaction with ^64^Cu was completed under similar conditions, in a decay-corrected radiochemical yield of 7%. Both [^68^Ga]Ga-DOTA-PEG_3_-TZ(PEG_4_-Octr)-PEG_2_-Trz-PEG_3_-Val-Cit-pABOC-FTY720 and [^64^Cu]Cu-DOTA-PEG_3_-TZ(PEG_4_-Octr)-PEG_2_-Trz-PEG_3_-Val-Cit-pABOC-FTY720 were assessed by radio-HPLC with ~95% radiochemical purity (Figures S13 and S14) for in vitro and in vivo evaluations.

### 3.3. In Vitro Assays of DOTA-PEG_3_-TZ(PEG_4_-Octr)-PEG_2_-Trz-PEG_3_-Val-Cit-pABOC-FTY720 

#### 3.3.1. SSTR2-Selective Cell Uptake and SSTR2-Mediated Internalization of [^64^Cu]Cu- DOTA-PEG_3_-TZ(PEG_4_-Octr)-PEG_2_-Trz-PEG_3_-Val-Cit-pABOC-FTY720

**Figure 2:**
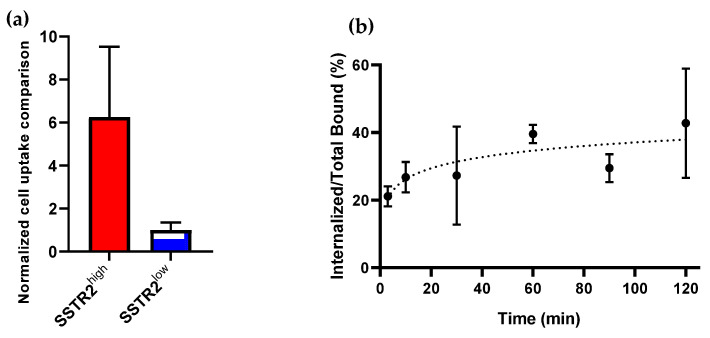
In vitro cell assays of [^64^Cu]Cu-DOTA-PEG_3_-TZ(PEG_4_-Octr)-PEG_2_-Trz-PEG_3_-Val-Cit-pABOC-FTY720: (**a**) Normalized SSTR2-selective uptake of [^64^Cu]Cu-DOTA-PEG_3_-TZ(PEG_4_-Octr)-PEG_2_-Trz-PEG_3_-Val-Cit-pABOC-FTY720 (uptake in SSTR2^low^ cells was set at 1). (**b**) Internalization of [^64^Cu]Cu-DOTA-PEG_3_-TZ(PEG_4_-Octr)-PEG_2_-Trz-PEG_3_-Val-Cit-pABOC-FTY720 in SSTR2^high^ AR42J cells. Data presented as average counts ± s.d. (n = 3).

With the functionality of PEG_4_-Octreotide, the construct was designed to maintain the desired SSTR2-selective binding and SSTR2-mediated internalization. The comparative in vitro assays were performed using the SSTR2^high^ AR42J cell line [40] and SSTR2^low^ PC3-Flu cells. As shown in Figure 2a, the normalized uptake of [^64^Cu]Cu-DOTA-PEG_3_-TZ(PEG_4_-Octr)-PEG_2_-Trz-PEG_3_-Val-Cit-pABOC-FTY720 demonstrated a six-fold higher accumulation in SSTR2^high^ AR42J cells than that in SSTR2^low^ PC3-Flu cells after 1 h of incubation, confirming the desired SSTR2-selective binding of the conjugate. Furthermore, incubation with SSTR2^high^ AR42J cells showed that approximately 40% of [^64^Cu]Cu-DOTA-PEG_3_-TZ(PEG_4_-Octr)-PEG_2_-Trz-PEG_3_-Val-Cit-pABOC-FTY720 became internalized after 60 min (Figure 2b). 

#### 3.3.2. Cathepsin-B-Mediated Release of FTY720 from DOTA-PEG_3_-TZ(PEG_4_-Octr)-PEG_2_-Trz-PEG_3_-Val-Cit-pABOC-FTY720

To assess whether the construct was capable of controlled release of FTY720 upon cathepsin-B-catalyzed cleavage, a release assay was performed using a previously reported procedure [41]. After 1 h of incubation at 37 °C, a sample was removed from the mixture, and analysis with LC/MS showed a molecular weight of 366.0488 [M + 2H_2_O + Na]^+^, which represents the molecular weight of FTY720 (307.2511 g/mol), two water molecules, and a sodium ion (Figures S15 and S16). The cathepsin-B-mediated release of FTY720 was further confirmed with a control assay, where the peak of 366.0488 was absent when the experiment was conducted under the same conditions in the absence of cathepsin B (Figure S17).

#### 3.3.3. The Cytotoxicity of FTY720 Was Masked by Its Conjugation with the Cathepsin B Prodrug Linkage, Val-Cit

**Figure 3:**
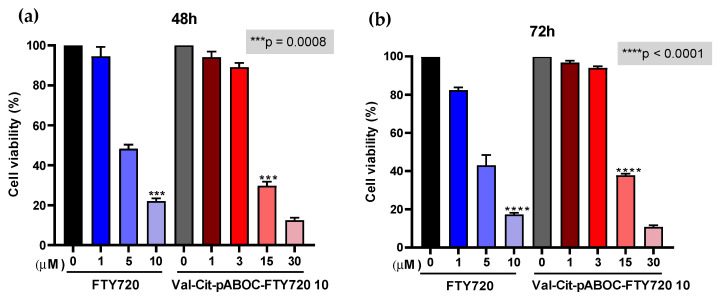
Cytotoxicity studies using PC3 cells incubated with different concentrations of FTY720 and Val-Cit-pABOC-FTY720 after (**a**) 48 h and (**b**) 72 h of treatment. Data are presented as average counts ± s.d. (n = 3) (unpaired *t*-tests performed using GraphPad Prism 9.4.1).

**Figure 4:**
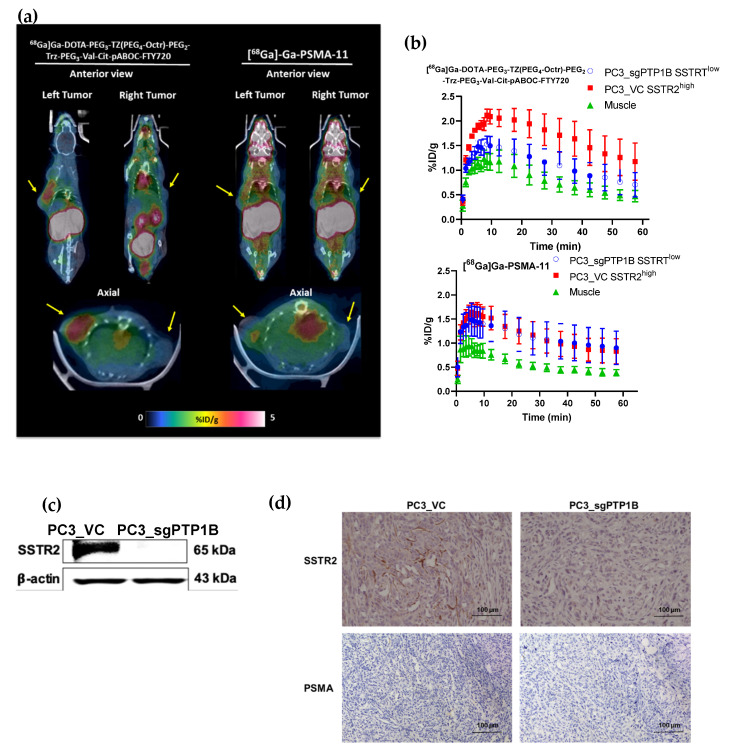
(**a**) Representative PET/CT images of SCID/NOD mice bearing SSTR2^high^ PC3_VC and SSTR2^low^ PC3_sgPTP1B xenografts during a 60-min dynamic PET scan with [^68^Ga]Ga-DOTA-PEG_3_-TZ(PEG_4_-Octr)-PEG_2_-Trz-PEG_3_-Val-Cit-pABOC-FTY720 (left) and [^68^Ga]Ga-PSMA-11 (right). Yellow arrows indicate the tumors. (**b**) Quantitative PET imaging analysis in tissues of interest. (**c**) Western blot showing high expression of SSTR2 in PC3_VC but low expression in PC3_sgPTP1B (β-actin as a loading control). (**d**) IHC staining of the excised tumor sections to assess the presence of SSTR2 and PSMA in the tumors. Data are presented as the average %ID/g ± s.d. (n = 4) (unpaired *t*-tests performed by GraphPad Prism 9.4.1).

Cell viability assays were performed using different concentrations of Val-Cit-pABOC-FTY720 and free FTY720 to compare their cytotoxicity. To that end, four different concentrations of the two compounds were used (0, 1, 5, and 10 µM), and the cells were incubated for either 48 or 72 h. The results showed that the EC_50_ for FTY720 was ~5 µM after 48 h (Figure 3a, blue bars) and 72 h (Figure 3b, blue bars) of incubation, while the EC_50_ for Val-Cit-pABOC-FTY720 was found to be >15 µM after 48 h (Figure 4a, red bars) and 72 h of treatment (Figure 4b, red bars), indicating that the prodrug Val-Cit-pABOC-FTY720 moiety had become three times less toxic than FTY720 itself. This implies that the side effects of free FTY720 can be mitigated by the prodrug design [42]. 

### 3.4. In Vivo Evaluation of [^68^Ga]Ga-DOTA-PEG_3_-TZ(PEG_4_-Octr)-PEG_2_-Trz-PEG_3_-Val-Cit-pABOC-FTY720

#### PET Imaging of Mice Showed That [^68^Ga]Ga-DOTA-PEG_3_-TZ(PEG_4_-Octr)-PEG_2_-Trz-PEG_3_-Val-Cit-pABOC-FTY720 had SSTR2-Selective Tumor Uptake and It Was Mainly Excreted through the Kidneys

Imaging studies were conducted in SCID/NOD mice bearing subcutaneously implanted PC3_VC cells (wild-type PC3 with high SSTR2 expression) in the left shoulder and PC3_sgPTP1B cells (PTP1B-gene-silenced PC3 cell with low SSTR2 expression) in the right shoulder. Clearly, the SSTR2^high^ PC3_VC tumor showed significantly higher accumulation of [^68^Ga]Ga-DOTA-PEG_3_-TZ(PEG_4_-Octr)-PEG_2_-Trz-PEG_3_-Val-Cit-pABOC-FTY720 throughout the duration of dynamic imaging (0–60 min) than the SSTR2^low^ PC3_sgPTP1B tumor (Figure 4a—Left). Quantitative imaging analysis revealed that the SSTR2^high^ tumor uptake peaked (2.1 ± 0.3 %ID/g) at 13 min post-injection (p.i.); in contrast, the SSTR2^low^ tumor uptake (1.5 ± 0.4 %ID/g) was at the same level as the muscle (Figure 4b—upper panel). The substantial accumulation of [^68^Ga]Ga-DOTA-PEG_3_-TZ(PEG_4_-Octr)-PEG_2_-Trz-PEG_3_-Val-Cit-pABOC-FTY720 in the kidneys, and then in the bladder, demonstrated that the radiolabeled conjugate was primarily cleared from the kidneys, which is the desired route of excretion of therapeutic small-molecule conjugates. 

The same mice were imaged using [^68^Ga]Ga-PSMA-11, and the results showed low uptake in both SSTR2^high^ PC3-VC and SSTR2^low^ PC3_sgPTP1B tumors (Figure 4a—right). In addition, [^68^Ga]Ga-PSMA-11 failed to differentiate the two tumor phenotypes (Figure 4b—lower panel) that are implicated in the progression of prostate cancer to NEPC. Taken together, these results suggest the potential of using [^68^Ga]Ga-DOTA-PEG_3_-TZ(PEG_4_-Octr)-PEG_2_-Trz-PEG_3_-Val-Cit-pABOC-FTY720 for noninvasive imaging detection of NEPC, and the T-SMPDC system could deliver FTY720 selectively to NEPC for targeted therapy. As shown in Figure 4c, the levels of SSTR2 expression in PC3_VC and PC3_sgPTP1B cells were assessed by Western blot (note: the original raw Western blots are presented in Figure S18). In addition, the excised PC3_VC and PC3_sgPTP1B tumors were confirmed to express SSTR2^high^ and SSTR2^low^ (Figure 4d—upper panel), respectively, by IHC. Furthermore, no appreciable level of PSMA expression was found in either of the tumor phenotypes (Figure 4d—lower panel). These observations (Figure 4c,d) further confirm our imaging findings with [^68^Ga]Ga-DOTA-PEG_3_-TZ(PEG_4_-Octr)-PEG_2_-Trz-PEG_3_-Val-Cit-pABOC-FTY720 and [^68^Ga]Ga-PSMA-11 (Figure 4a,b). 

## 4. Discussion

Targeted therapy serves as the foundation of precision medicine. According to the latest information provided by the National Cancer Institute [43], most targeted cancer therapies currently utilize either small-molecule drugs or monoclonal antibodies. Small molecule drugs are typically developed for intracellular targets, as they are small and can be readily engineered to cross the plasma membrane. As such, for actions on molecular targets that are highly upregulated in cancer cells, targeted cancer therapies have demonstrated significant progression-free survival (PFS) benefits over conventional chemotherapies, along with reduced off-target toxicity and side effects [44]. However, many of the molecular targets or pathways that have been explored for targeted therapies are not absent in normal cells; instead, they are tightly regulated, such as Sphk1. To achieve the goal of NEPC-targeted delivery and controlled intracellular release of an SphK1 inhibitor to suppress the function of Sphk1, which is implicated in the progression of CRPC to NEPC [20,35,45], our design strategy of T-SMPDC takes advantage of the chemistry of 2,4,6-trichloro-1,3,5-triazine for modular assembly of different functional components.

Similar to the synthesis of our recently reported T-SMPDC [20], the modular assembly on the trifunctional platform started from stepwise chemical modifications of each functional component. To conjugate the prodrug moiety, Val-Cit-pABOC-FTY720, we found that it was necessary to add an extra spacer between the prodrug moiety and the 1,3,5-triazine core to minimize the steric hindrance, thereby facilitating the reaction. Among the many reported strategies available, we took a click chemistry route by functionalizing 2,4,6-trichloro-1,3,5-triazine with propargyl-PEG2-amine and adding an azido-PEG to Val-Cit-pABOC-FTY720. The click chemistry reaction was straightforward to form TZ-PEG_2_-Trz-PEG_3_-Val-Cit-pABOC-FTY720. In addition, it minimized the potential interferences of various functional groups in following the modular assembly of the T-SMPDC. The yields of the four major synthetic steps towards DOTA-PEG_3_-TZ(PEG_4_-Octr)-PEG_2_-Trz-PEG_3_-Val-Cit-pABOC-FTY720 were ~45% (for compound **10**), ~54% (compound **11**), ~11% (compound **12**), and ~33% (compound **13**), which are higher than what we reported for the synthesis of our first T-SMPDC [20]. This demonstrates the synthetic capability that we have developed to scale up the synthesis of various T-SMPDC systems when translational studies are warranted to proceed. 

The radiolabeling of DOTA-PEG_3_-TZ(PEG_4_-Octr)-PEG_2_-Trz-PEG_3_-Val-Cit-pABOC-FTY720 with ^68^Ga or ^64^Cu was straightforward. However, the radiochemical yield was low, because it was calculated based on the radioactivity of ^68^Ga or ^64^Cu added to a fixed amount of the conjugate. Under these radiochemical conditions, we were able to achieve an optimal molar activity (38 GBq/µmol) for [^68^Ga]Ga-DOTA-PEG_3_-TZ(PEG_4_-Octr)-PEG_2_-Trz-PEG_3_-Val-Cit-pABOC-FTY720 to be used for in vivo evaluations in tumor-bearing mouse models, where the highest achievable molar activity is desired. However, we acknowledge that the radiochemical reaction conditions can be further optimized to improve the radiochemical yield as well as the molar activity. It should be noted that the incorporation of DOTA into the T-SMPDC can also potentiate the use of the prodrug conjugate systems for developing targeted radiotheranostic agents when loaded with β- or α-emitters. 

To achieve NEPC-targeted delivery and controlled release of an intracellular target therapy, we chose an octreotide molecule to target SSTR2, whose expression is arguably elevated as CRPC progresses to NEPC. In addition, SSTR2 can mediate the desired internalization of the T-SMPDC upon its ligand binding of octreotide. Although debates on the correlation of SSTR2 with NEPC exist in the literature, we believe that the main reason perhaps arises from the heterogeneous and dynamic nature of the innervated tumor microenvironment (TME) [23], which makes biopsy-based IHC results either inaccurate or unrepresentative [46]. The choice of SSTR2 as the target for our T-SMPDC design does not reflect our position in these debates; instead, it reflects the fact that the innervated TME remains to be further explored. Even if it is ultimately confirmed that SSTR2 is not among the specific markers of NEPC, the design concept of T-SMPDC and its modular synthesis presented in this work can be readily adapted to accommodate the rapid advances in the field of cancer innervation. 

The proof-of-concept biological studies demonstrated that the T-SMPDC can initiate a substantial extent (~40%) of SSTR2-mediated internalization for intracellular delivery of the whole conjugate. The prodrug design was able to release FTY720 intact via cathepsin B. Importantly, the cytotoxicity of FTY720 can be masked when conjugated to mitigate the potential side effects of using FTY720 for cancer therapy. 

The two tumor models used in the small animal imaging evaluation were established by wild-type PC3 cells with high expression of SSTR2, and by PTP1B-silenced PC3 cells (PC3_sgPTP1B) with low expression of SSTR2. It should be noted that PTP1B, protein tyrosine phosphatase non-receptor 1, is a soluble endoplasmic-reticulum-associated tyrosine-specific phosphatase. It plays a well-established role in insulin signaling [47,48]. To date, it has been considered as a therapeutic target for type 2 diabetes and cancer [24,25]. The dynamic PET imaging with [^68^Ga]Ga-DOTA-PEG_3_-TZ(PEG_4_-Octr)-PEG_2_-Trz-PEG_3_-Val-Cit-pABOC-FTY720 clearly showed that the radiolabeled agent was capable of differentiating the two phenotypes of PC3 xenografts by their distinct expressions of SSTR2, further validating our design concept of the T-SMPDC. Encouragingly, the T-SMPDC displayed a desirable in vivo kinetic profile, with clearance from the kidneys. With regards to the use of the T-SMPDC for therapeutic evaluation, we can further optimize its structure by varying the linkages to enhance the tumor uptake and retention of the conjugate, as well as improving the contrast ratios of target/non-target tissues. 

To date, we have witnessed the paradigm-shifting roles of PSMA-targeted radiopharmaceuticals in prostate cancer patient care, as represented by [^68^Ga]Ga-PSMA-11 and [^18^F]F-DCFPyl for PET imaging and [^177^Lu]Lu-PSMA-617 for radiotherapy [6]. However, not every prostate cancer phenotype has PSMA expression [49]. Indeed, during the progression of CRPC to NEPC, the expression of PSMA becomes attenuated, limiting the benefits of PSMA-targeted radiopharmaceuticals to the patient populations with non-PSMA-expressing tumors or metastases. As clearly demonstrated in our small animal imaging evaluation (Figure 4), [^68^Ga]Ga-PSMA-11 failed to detect either SSTR2^high^ PC3-VC or SSTR2^low^ PC3_sgPTP1B tumors, due to their lack of PSMA expression. This fact necessitates the development of radio- and chemotheranostic agents by targeting other molecular mechanisms, such as the T-SMPDC presented in this work. Indeed, many other genes and proteins have been reported in association with NEPC [50], and recently we have validated the synaptic vesicle glycoprotein 2 isoform A (SV2A) [23] as a marker of NEPC.

It is noteworthy that FTY720 is an Sphk1 inhibitor that is approved by the United States Food and Drug Administration (US-FDA) for the treatment of multiple sclerosis. Repurposing of FTY720 for cancer therapies is now only reported in preclinical studies [24,51]. It remains to be seen whether SphK1 could serve as a practical target to develop NEPC therapies. However, we are not limited to Sphk1. As new druggable intracellular targets will certainly emerge in future, we will be able to readily incorporate them into the modular design of our T-SMPDC system.

## 5. Conclusions

We successfully designed, synthesized, and evaluated a T-SMPDC system, DOTA-PEG_3_-TZ(PEG_4_-Octr)-PEG_2_-Trz-PEG_3_-Val-Cit-pABOC-FTY720, for NEPC-targeted delivery and controlled intracellular release of an Sphk1 inhibitor to suppress the function of Sphk1, which is implicated in the progression of CRPC to NEPC. The T-SMPDC system offers advantages over ADCs in terms of in vivo pharmacokinetics, solid tumor penetration, definitive chemical structure, and ease of synthesis. In addition, the modular assembly feature of our accomplished synthesis allows a rapid adaption of the T-SMPDC platform to target other intracellular oncotargets or to deliver other potent chemotherapy molecules as necessary.

## Data Availability

Data are contained within the article.

## Supplementary Materials

The following supporting information can be downloaded at: https://www.mdpi.com/article/10.3390/pharmaceutics15020481/s1, Figure S1. MS (ESI) of DOTA-PEG_3_ **2**; Figure S2. MS (ESI) of PEG2-Trz **4**; Figure S3. HPLC chromatogram of PEG_2_-Trz **4**; Figure S4. MS (ESI) of PEG_4_-Octr **8**; Figure S5. HPLC chromatogram of PEG_4_-Octr **8**; Figure S6. MS (ESI) of Val-Cit-pABOC-FTY720 **10**. MS (ESI) *m/z* calcd: 941.5586; found: 942.4480 ([M + H]^+^); Figure S7. HPLC chromatogram of Val-Cit-pABOC-FTY720 **10**; Figure S8. MS (ESI) of TZ-PEG_2_-Trz-PEG_3_-Val-Cit-pABOC-FTY720 **11**. MS (ESI) *m/z* calcd: 1231.5923; found: 1232.61255 [M + H]^+^; Figure S9. MS (ESI) of DOTA-PEG_3_-TZ-PEG_2_-Trz-PEG_3_-Val-Cit-pABOC-FTY720 **12**. MS (ESI) *m/z* calcd: 1970.1623; found: 1971.9273 ([M + H]^+^); Figure S10. HPLC chromatogram of DOTA-PEG_3_-TZ-PEG_2_-Trz-PEG_3_-Val-Cit-pABOC-FTY720 **12**; Figure S11. MALDI-TOF spectra of DOTA-PEG_3_-TZ(PEG_4_-Octr)-PEG_2_-Trz-PEG_3_-Val-Cit-pABOC-FTY720 **13**. MALDI-TOF *m/z* calcd: 3031.5803; found: 1517.9304 [M + 2H]^+2^; Figure S12. HPLC chromatogram of DOTA-PEG_3_-TZ(PEG_4_-Octr)-PEG_2_-Trz-PEG_3_-Val-Cit-pABOC-FTY720 **13**; Figure S13. HPLC chromatogram of [^68^Ga]Ga- DOTA-PEG_3_-TZ(PEG_4_-Octr)-PEG_2_-Trz-PEG3-Val-Cit-pABOC-FTY720; Figure S14. HPLC chromatogram of [^64^Cu]Cu- DOTA-PEG_3_-TZ(PEG_4_-Octr)-PEG_2_-Trz-PEG3-Val-Cit-pABOC-FTY720; Figure S15. MS (ESI) of cathepsin B treated compound **13**; Figure S16. MS (ESI) of cathepsin B treated compound **13** (Enlarged area of the peaks of interest); Figure S17. MS (ESI) of cathepsin B untreated compound **13**; Figure S18. Original western blots for Figure 4c. sgNT: PC3_VC; CR1: PC3_sgPTP1B (CRISPR1 knockout of PTP1B); CR2: another clone of PC3_sgPTP1B; M: protein marker; β-action: loading control. References [52,53] are cited in the supplementary materials.
