# Supplementary material for: A Theranostic Small-Molecule Prodrug Conjugate for Neuroendocrine Prostate Cancer
Source: Pharmaceutics. 2023 Feb 1;15(2):481. doi: 10.3390/pharmaceutics15020481 (PMC9967013; doi:10.3390/pharmaceutics15020481)
Supplement: Supplementary file 1 [file pharmaceutics-15-00481-s001.zip › pharmaceutics-2168735-supplementary.pdf]

Supplementary Materials

Chemistry

Synthesis of tri-tert-butyl 2,2′,2′′-(10-(16-amino-2-oxo-7,10,13-trioxa-3-azahexadecyl)-1,4,7,10-tetraazacyclododecane-1,4,7-triyl)triacetate (DOTA-PEG$_3$) **2**

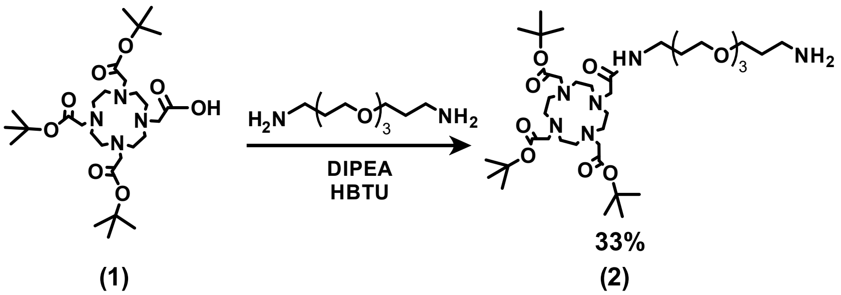

33%

(1) (2)

3,3'-((oxybis(ethane-2,1-diyl))bis(oxy))bis(propan-1-amine) (32 µL, 145 µmol) was added to a 1.5 ml of DCM at 4°C followed by the addition of 26µL (149 µmol) of DIPEA and stirred for approximately 5 min. In a separated vial the Tri-tert-butyl 1,4,7,10-tetraazacyclododecane-1,4,7,10-tetraacetate (100 mg, 175 µmol), and HBTU (66mg, 174 µmol) was dissolved in 1 ml of DCM, followed by the dropwise addition of the solution of the diamine. The reaction mixture was left to stir for 12 hrs. The product was purified using HPLC (0 min: 10% H_2O, 90% CH_3CN; 15 min: 20% H_2O 80% CH_3CN; 20 min: 10% H_2O 90% CH_3CN, flow of 20 mL/min all solvents contained 0.1% TFA) Obtained 44 mg (yield 33%) of a tacky oil. The product was confirmed by mass spectrometry (Figure S1). MS (ESI) m/z calcd for $C_{38}H_{74}N_6O_{10}$: 774.5466; found: 387.3141 ($[M+2H]^{+2}$).

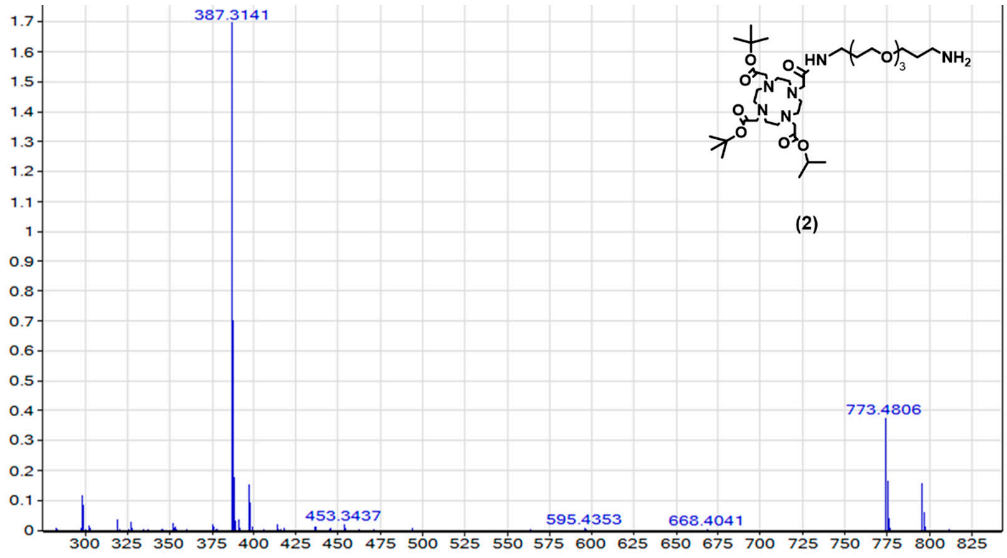

Figure S1. MS (ESI) of DOTA-PEG$_3$ **2**

Synthesis of 4,6-dichloro-N-(2-(2-(prop-2-yn-1-yloxy)ethoxy)ethyl)-1,3,5-triazin-2-amine (PEG2-Trz) ***4***

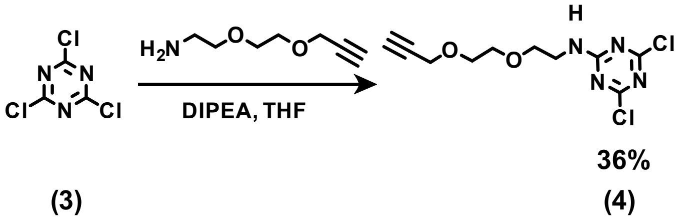

2,4,6-Trichloro-1,3,5-triazine (92 mg, 1.0 mmol) was dissolved in THF (15 mL) and stirred under nitrogen for 5 min. To this solution DIPEA (96 μL, 0.55 mmol) was added under nitrogen at 0 °C and stirred for another 10 minutes. A solution of propargyl-PEG2-amine (71 mg, 0.5 mmol) in THF (4mL) was added dropwise to this solution at 0 °C and stirring was continued for 3 hrs. The reaction mixture was concentrated under vacuum to afford the crude product, which was then purified by a reversed phase HPLC (0 min: 20% CH_3CN, 80% H_2O; 18 min: 80% CH_3CN, 20% H_2O, flow of 20 mL/min, all solvents contained 0.1% TFA). Pure fractions from HPLC were combined and lyophilized to give a white solid compound (52 mg, yield 36%). The product was confirmed by mass spectrometry (Figure S2). MS (ESI) m/z calcd for $C_{10}H_{12}Cl_2N_4O_2$: 290.0337; found: 291.0376 ($[M + H]^+$). 1H NMR (400 MHz, $CDCl_3$): δ 6.48 (s, 1H), 4.21 (s, 2H), 3.77-3.57 (m, 8H), 2.46 (S, 1H). The purity was assessed by HPLC (Figure S3).

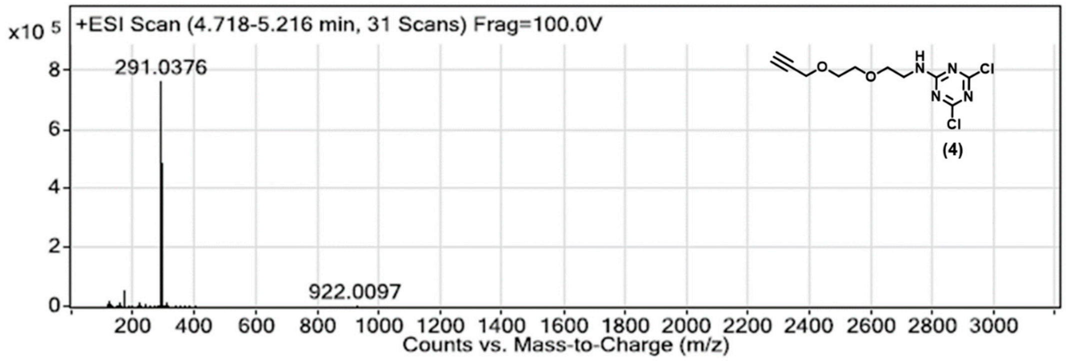

Figure S2. MS (ESI) of PEG2-Trz **4**

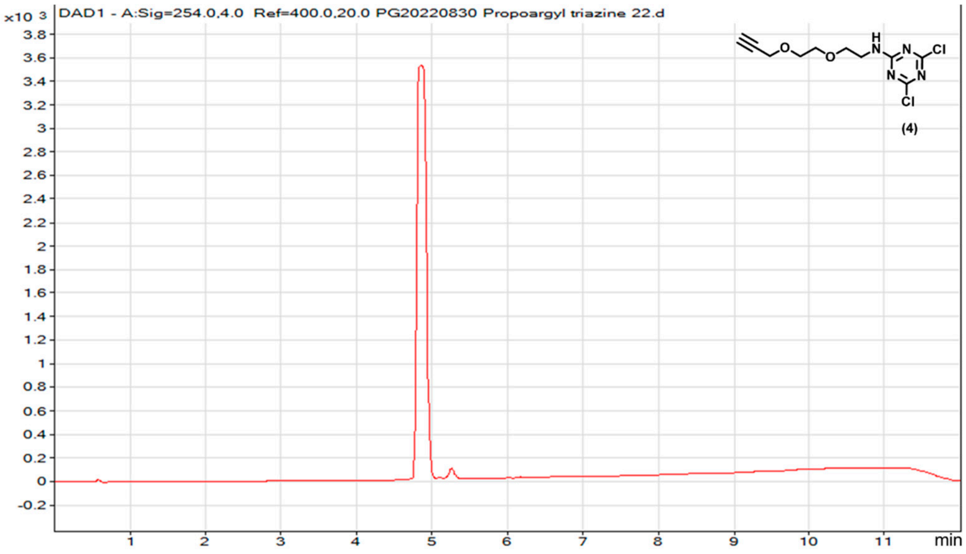

Figure S3. HPLC chromatogram of PEG2-Trz **4**

Synthesis of 2,5-dioxopyrrolidin-1-yl 2,2-dimethyl-4-oxo-3,8,11,14,17-pentaoxa-5-azaicosan-20-oate ***6***

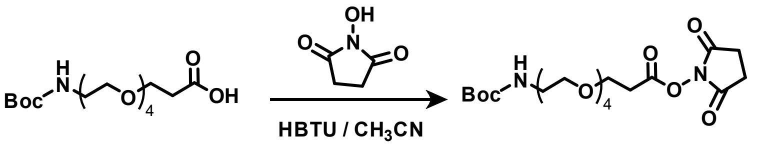

(5) (6)

In a glass reaction vial 2,2-dimethyl-4-oxo-3,8,11,14,17-pentaoxa-5-azaicosan-20-oic acid **(5)** (275 mg, 0.75 mmol.), 1-hydroxypyrrolidine-2,5-dione (86 mg, 0.75 mmol) and HBTU (392 mg, 1.03 mmol) were dissolved in acetonitrile and stirred at r.t for two hrs. The solvent was evaporated and the product was used as obtained for the next reaction.

Synthesis of ((4R,10R,13S,16S,19R)-10-((1H-indol-3-yl)methyl)-4-((R)-2-(1-amino-3,6,9,12-tetraoxapentadecan-15-amido)-3-phenylpropanamido)-13-(4-aminobutyl)-7-benzyl-16-((R)-1-hydroxyethyl)-5,8,11,14,17-pentaoxo-1,2-dithia-6,9,12,15-tetraazacycloicosane-19-carbonyl)-L-threonine (PEG4-Octr) 8

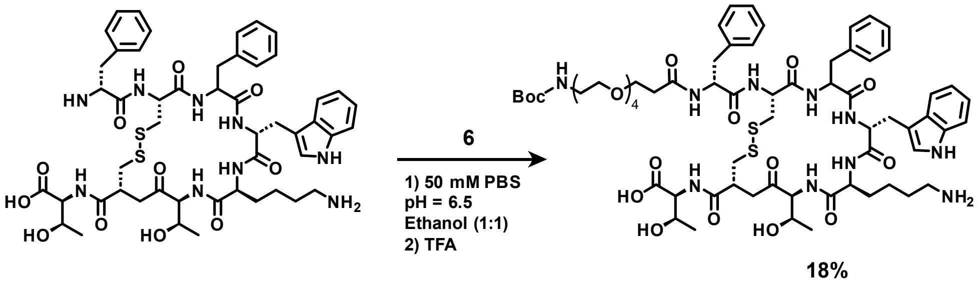

(7) (8)

Octreotide acetate **7** (4 mg, 4 µmol, 1 eq), was dissolved in a 1:1 solution of ethanol and 60 mM phosphate buffer (pH = 6.5) total volume 2 mL. Subsequently, 2,5-dioxopyrrolidin-1-yl 2,2-dimethyl-4-oxo-3,8,11,14,17-pentaoxa-5-azaicosan-20-oate, **6** (109 mg, 0.24 mmol, 60 eq) was added and the solution was

stirred at rt. overnight. The crude was purified by HPLC (0 min: 10% CH_3CN, 90% H_2O; 14 min: 50% CH_3CN, 50% H_2O; 19 min: 90% CH_3CN, flow of 20 mL/min all solvents contained 0.1% TFA) to obtain the desired product (983 μg, 18% yield), corroborated by mass spectrometry (Figure S4). MS (ESI) m/z calcd: 1365.6349; found: 1366.6574 $[M + H]^+$, 683.8326 ($[M + 2H]^{+2}$). The purity was assessed by HPLC (Figure S5).

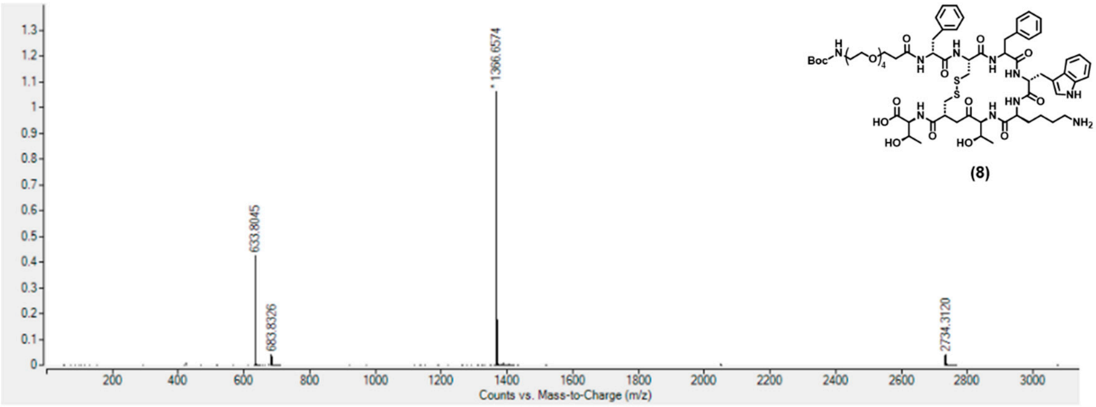

Figure S4. MS (ESI) of PEG4-Octr **8**

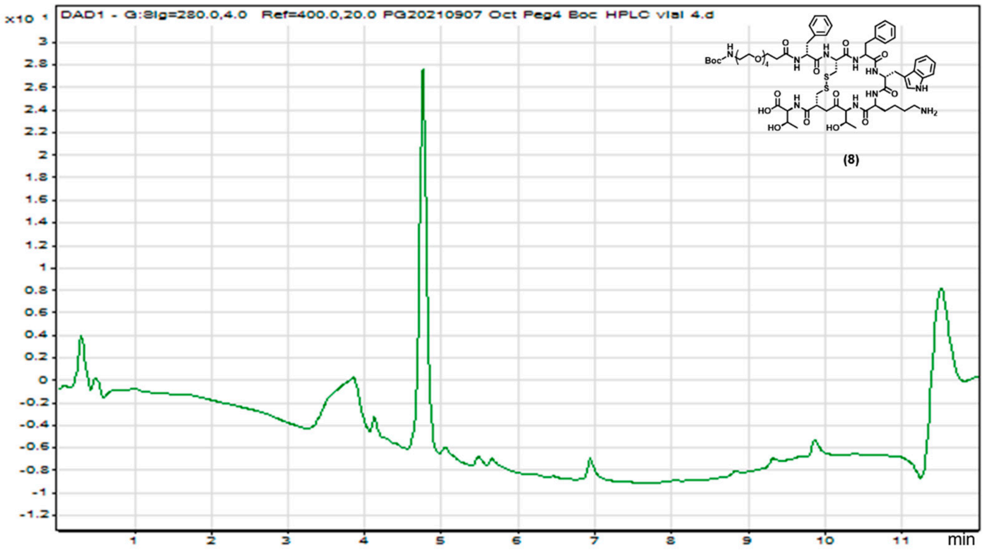

Figure S5. HPLC chromatogram of PEG4-Octr **8**

Characterization of Val-Cit-pABOC-FTY720 ***10***

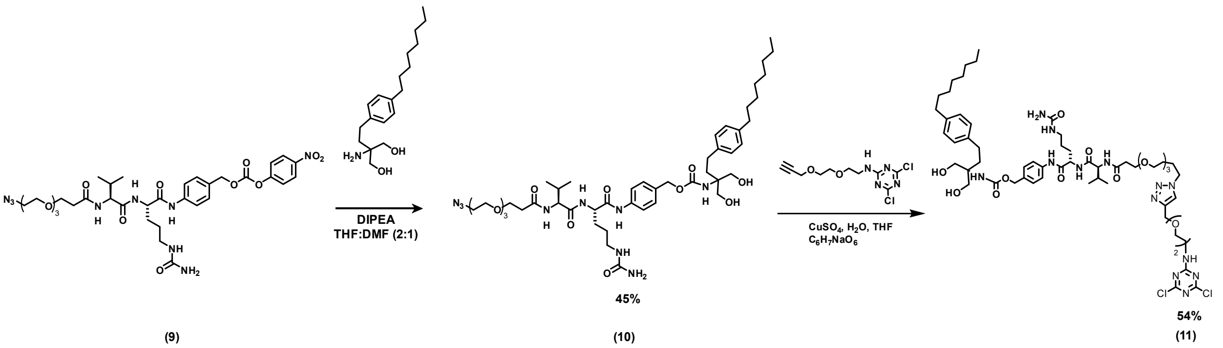

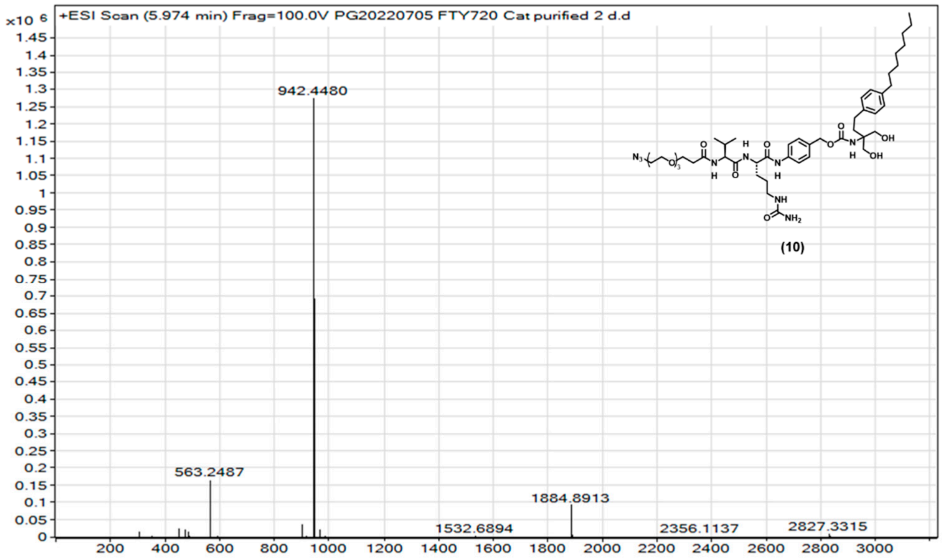

Figure S6. MS (ESI) of Val-Cit-pABOC-FTY720 **10.** MS (ESI) m/z calcd: 941.5586; found: 942.4480 ([M + H]$^+$)

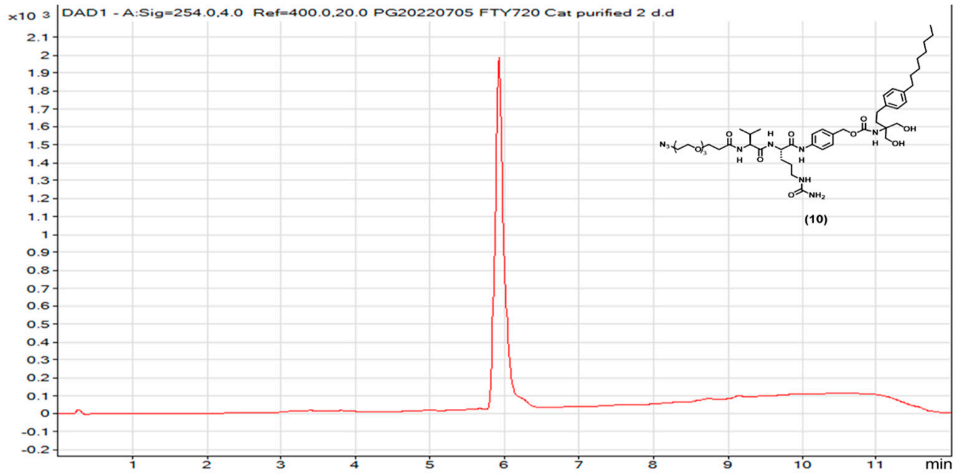

Figure S7. HPLC chromatogram of Val-Cit-pABOC-FTY720 **10**

Characterization of TZ-PEG2-Trz-PEG3-Val-Cit-pABOC-FTY720 **11**

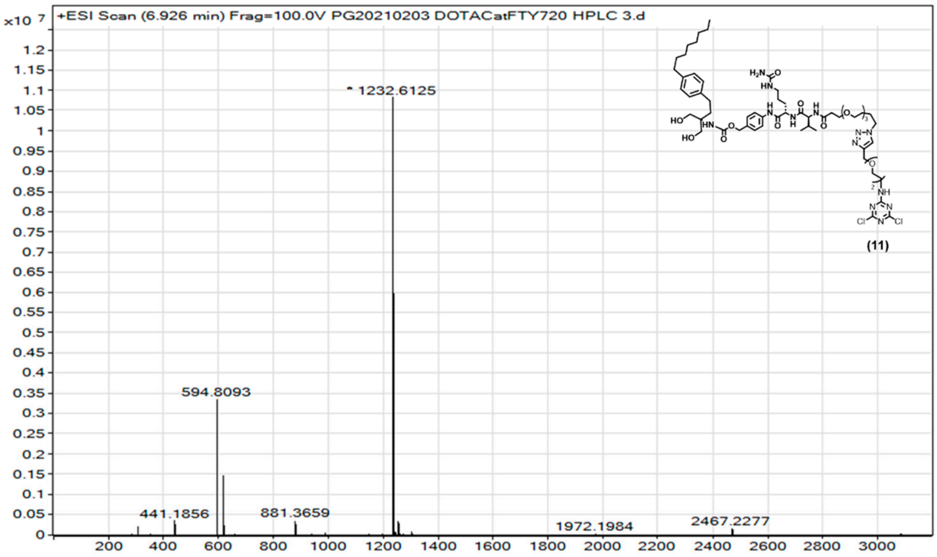

Figure S8. MS (ESI) of TZ-PEG2-Trz-PEG3-Val-Cit-pABOC-FTY720 **11.** MS (ESI) m/z calcd: 1231.5923; found: 1232.61255 $[M + H]^+$

Characterization of DOTA-PEG3-TZ-PEG2-Trz-PEG3-Val-Cit-pABOC-FTY720 **12**

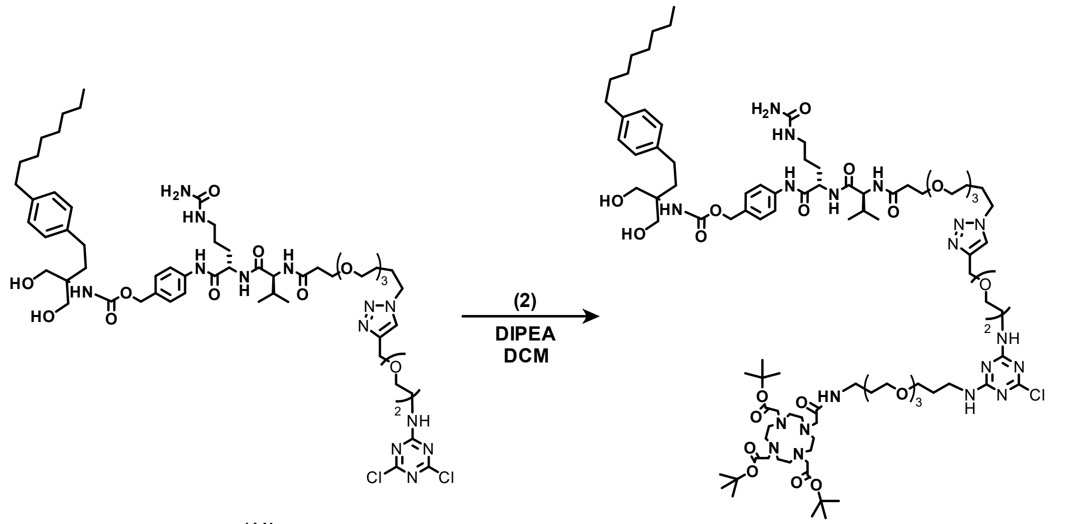

(11) **(12)**

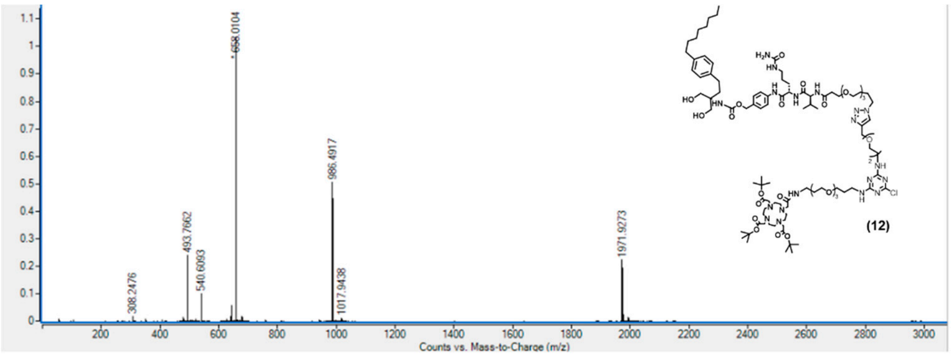

Figure S9. MS (ESI) of DOTA-PEG3-TZ-PEG2-Trz-PEG3-Val-Cit-pABOC-FTY720 **12.** MS (ESI) m/z calcd: 1970.1623; found: 1971.9273 ([M + H]+)

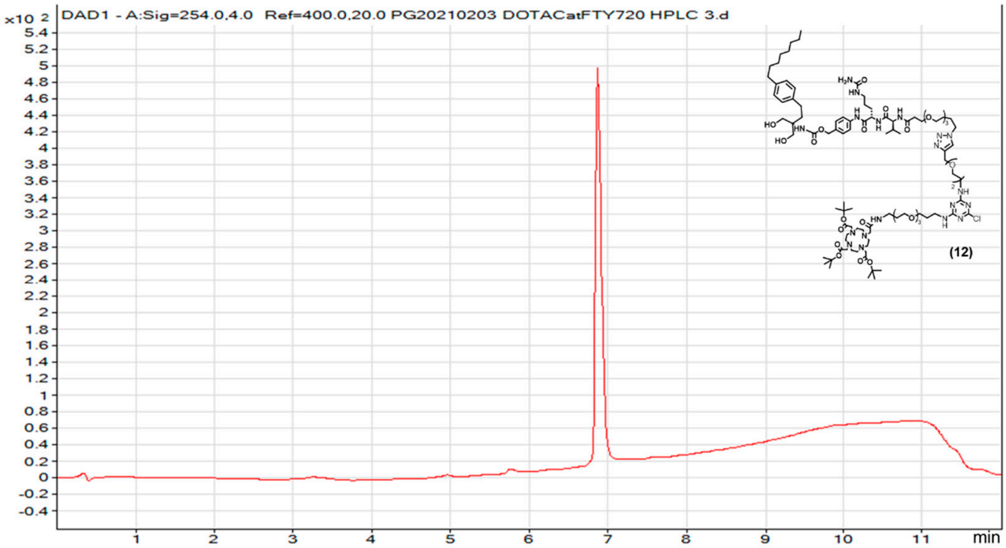

Figure S10. HPLC chromatogram of DOTA-PEG3-TZ-PEG2-Trz-PEG3-Val-Cit-pABOC-FTY720 **12**

Characterization of DOTA-PEG$_3$-TZ(PEG$_4$-Octr)-PEG$_2$-Trz-PEG$_3$-Val-Cit-pABOC-FTY720 ***13***

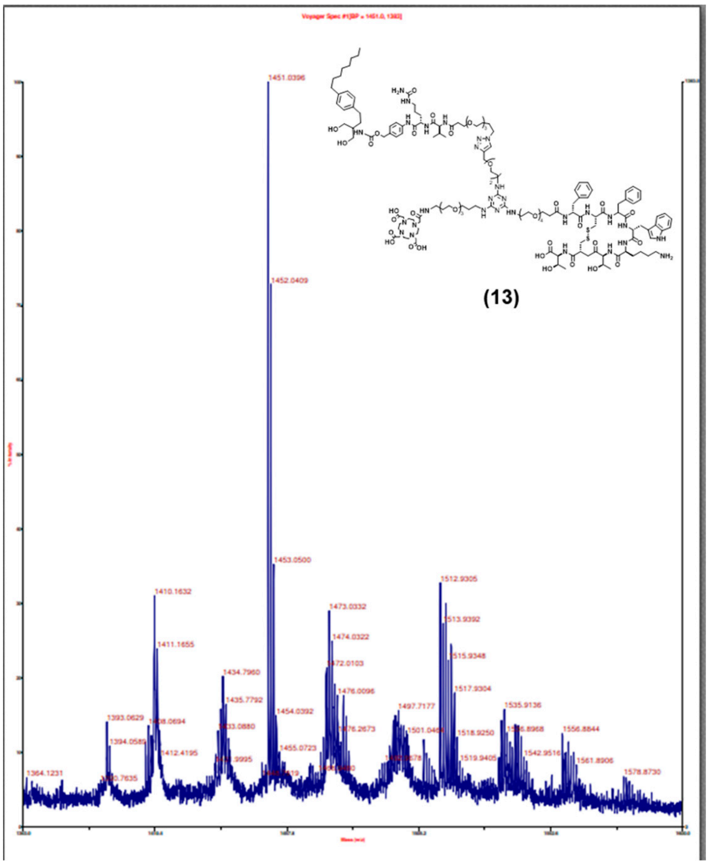

Figure S11. MALDI-TOF spectra of DOTA-PEG$_3$-TZ(PEG$_4$-Octr)-PEG$_2$-Trz-PEG$_3$-Val-Cit-pABOC-FTY720 **13.** MALDI-TOF m/z calcd: 3031.5803; found: 1517.9304 $[M + 2H]^{+2}$

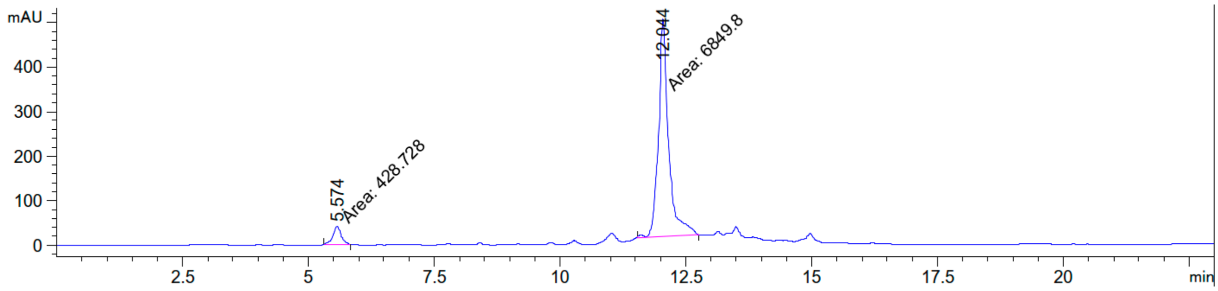

Figure S12. HPLC chromatogram of DOTA-PEG$_3$-TZ(PEG$_4$-Octr)-PEG$_2$-Trz-PEG$_3$-Val-Cit-pABOC-FTY720 **13**

Radiochemical purity of [^{68}Ga]Ga- DOTA-PEG$_3$-TZ(PEG$_4$-Octr)-PEG$_2$-Trz-PEG3-Val-Cit-pABOC-FTY720

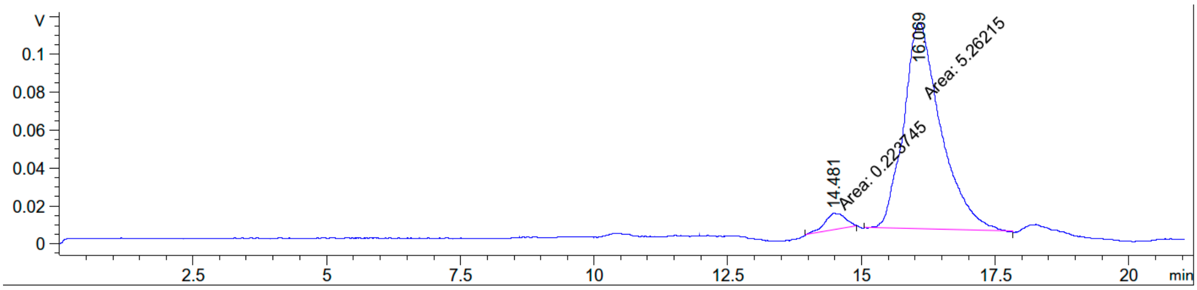

Figure S13. HPLC chromatogram of [^{68}Ga]Ga- DOTA-PEG$_3$-TZ(PEG$_4$-Octr)-PEG$_2$-Trz-PEG3-Val-Cit-pABOC-FTY720

Radiochemical purity of [^{64}Cu]Cu- DOTA-PEG$_3$-TZ(PEG$_4$-Octr)-PEG$_2$-Trz-PEG3-Val-Cit-pABOC-FTY720

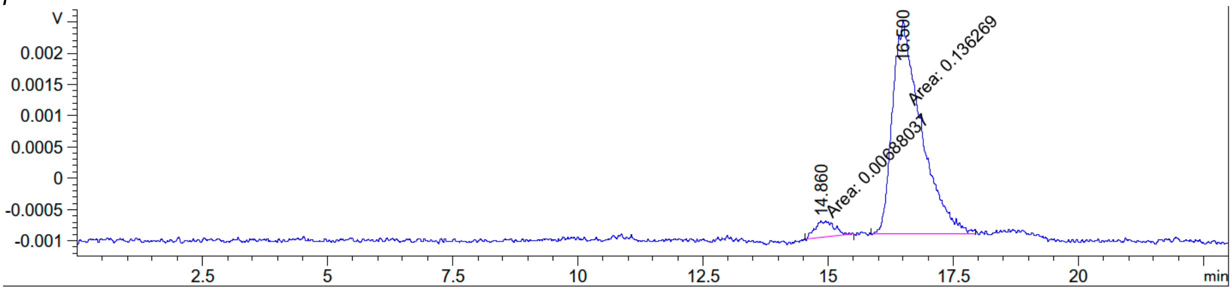

Figure S14. HPLC chromatogram of [^{64}Cu]Cu- DOTA-PEG$_3$-TZ(PEG$_4$-Octr)-PEG$_2$-Trz-PEG3-Val-Cit-pABOC-FTY720

Production of ^{68}Ga

A target solution of 1 M $^{68}Zn(NO_3)_2$ in 0.3 M nitric acid was prepared by slow dissolution of enriched ^{68}Zn oxide powder in nitric acid (trace metal basis), followed by dilution with TraceSELECT (or ultra-pure) water to achieve the desired concentration. The solution was then equilibrated for 4 h, followed by filtration into a vial. The target material was then irradiated on a General Electric PETtrace 880 cyclotron (Chicago, IL, USA) for 60 to 90 min at 14 MeV to produce $^{68}Ga(NO_3)_3$ from the $^{68}Zn(p,n)^{68}Ga$ reaction. The produced ^{68}Ga was purified using a GE FASTlab Synthesizer to obtain the ^{68}Ga chloride species [1,2].The purified gallium [^{68}Ga] chloride solution ([^{68}Ga]$GaCl_3$, approximately 1.9 GBq in 3-4 mL of 0.1 N HCl) was transferred to a 15 mL falcon tube for further purification. Equal volume of concentrated hydrochloric acid (3-4 mL) was added to the [^{68}Ga]$GaCl_3$ solution and vortexed for 15 seconds. To that, 1 mL of isopropyl ether was added and vortexed for 30 seconds. The aqueous layer (7-8 mL) was removed and discarded to waste. (Note: [^{68}Ga] is in the top organic layer, usually 0.9 MBq are extracted). To the organic layer, 150 µL of HEPES buffer (2 M, pH 7.5) was added and vortexed for 30 seconds, followed by addition of hydrochloric acid (70 µL, 0.6 M) and the solution was vortexed for 30 seconds. The aqueous layer (~220µL) containing the purified [^{68}Ga]$GaCl_3$ was transferred to a 2 mL eppendorf tube.

Production of ^{64}Cu

For the solid target electrodeposition, ^{64}Ni (200mg, Isoflex, San Francisco, Ca, USA) was dissolved in 10 ml trace metal nitric acid (stirring about 3 hrs at 80 ºC). Then, about 12 mL of 12.5 M sodium hydroxide was added to change nickel nitrate to nickel hydroxide. The nickel hydroxide was washed 5 times via centrifugation at 4500 rpm for 15 minutes. The washed nickel hydroxide was re-suspended in 10 mL of Millipore water, followed by the addition of ~250 µl sulfuric acid dropwise to dissolve nickel hydroxide and make nickel sulfate. After, ~200 µl boric acid was added to the nickel sulfate. Next, ~150 µl of 12.5 M sodium hydroxide was added dropwise to adjust the pH of solution to ~3.0-3.5. The solution (5 mL) was transferred to the electrodeposition cell and connected to the electrodes. The electrodeposition was ran at about 60-70 ºC with air agitation at about 3 mA for about 12 hrs. The gold disk containing the electrodeposited ^{64}Ni was irradiated on a General Electric PETtrace 880 cyclotron (Chicago, IL, USA) for 4 h at 15 MeV to produce ^{64}Cu from the $^{64}Ni(p,n)^{64}Cu$ reaction. The produced ^{64}Cu was purified using a Comecer Solid target automated system (Castel, Bologna, Italy), to obtain $^{64}CuCl_2$ in 0.1M HCl.

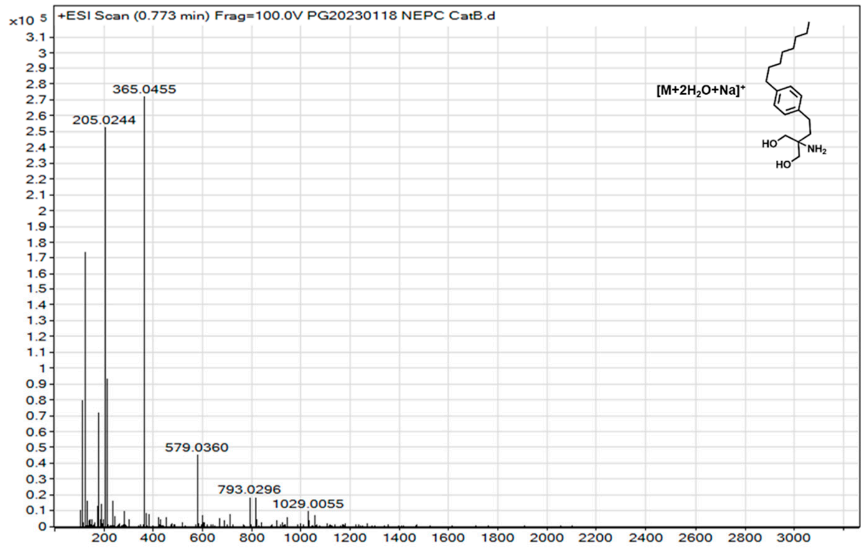

Figure S15. MS (ESI) of cathepsin B treated compound **13**

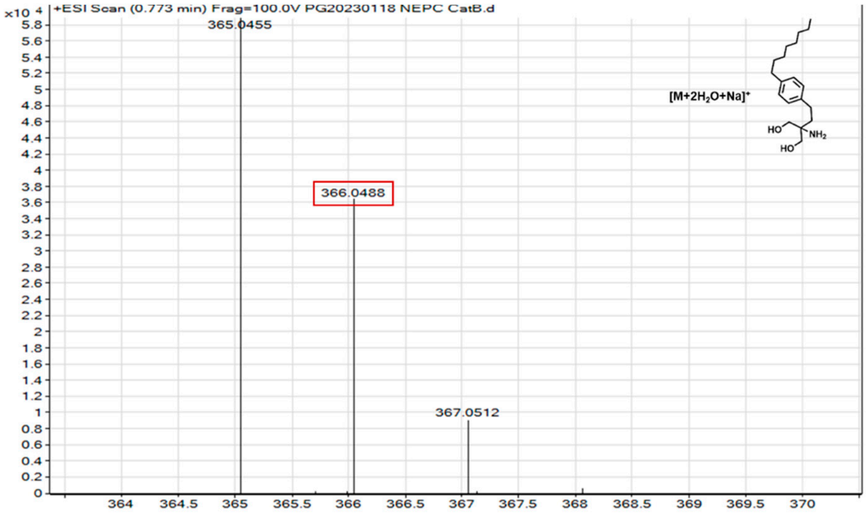

Figure S16. MS (ESI) of cathepsin B treated compound **13** (Enlarged area of the peaks of interest)

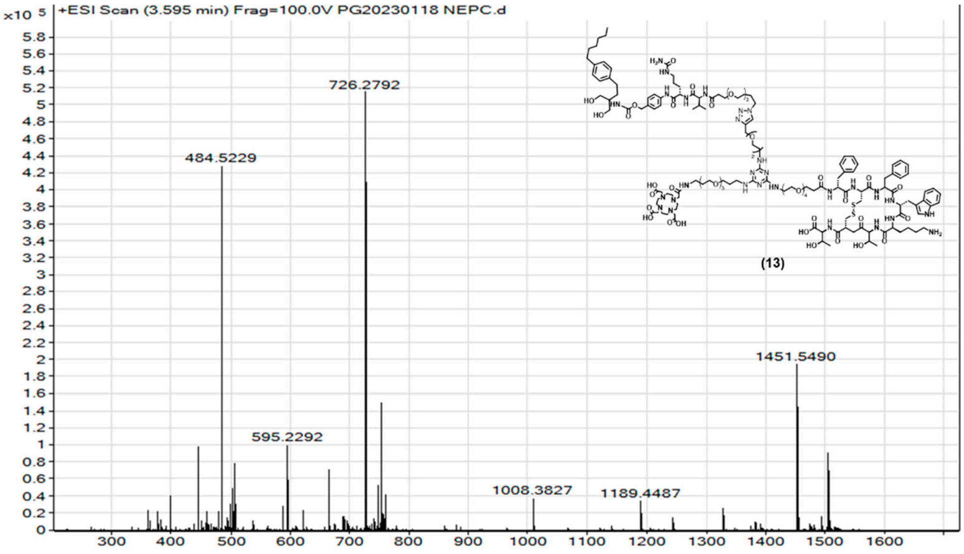

Figure S17. MS (ESI) of cathepsin B untreated compound **13**

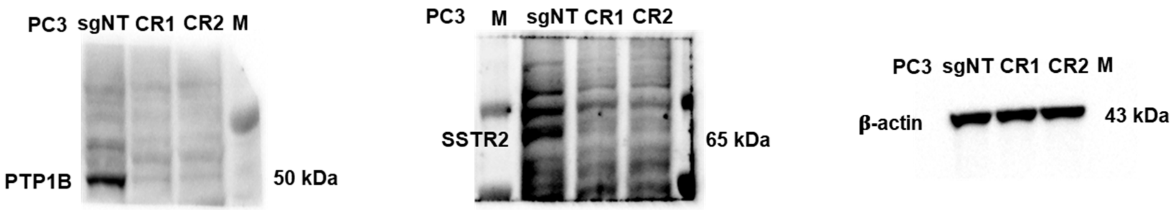

Figure S18. Original western blots for **Figure 4c**. sgNT: PC3_VC; CR1: PC3_sgPTP1B (CRISPR1 knockout of PTP1B); CR2: another clone of PC3_sgPTP1B; M: protein marker; β-action: loading control.

References

1. Mansi, R.; Abid, K.; Nicolas, G.P.; Del Pozzo, L.; Grouzmann, E.; Fani, M. A New 68Ga-Labeled Somatostatin Analog Containing Two Iodo-Amino Acids for Dual Somatostatin Receptor Subtype 2 and 5 Targeting. *EJNMMI Res.* **2020**, *10*, 90, doi:10.1186/s13550-020-00677-3.
2. Riga, S.; Cicoria, G.; Pancaldi, D.; Zagni, F.; Vichi, S.; Dassenno, M.; Mora, L.; Lodi, F.; Morigi, M.P.; Marengo, M. Production of Ga-68 with a General Electric PETtrace Cyclotron by Liquid Target. *Phys. Medica* **2018**, *55*, 116–126, doi:10.1016/j.ejmp.2018.10.018.
